# Comparison of SARS-CoV-2 seroconversion in children with chronic diseases with healthy children and adults during the first waves of the COVID-19 pandemic

**DOI:** 10.3389/fped.2023.1210181

**Published:** 2023-08-07

**Authors:** Levi Hoste, Agnieszka Prytula, Jo Dehoorne, Ruth De Bruyne, Stephanie Van Biervliet, Kathleen De Waele, Evelyn Maes, Victoria Bordon, Arnaud Vanlander, Karlien Claes, Johan Vande Walle, Petra Schelstraete, Sabine Van daele, Filomeen Haerynck

**Affiliations:** ^1^ Department of Pediatric Pulmonology, Infectious Diseases and Immunology, Ghent University Hospital, Ghent, Belgium; ^2^ Primary Immunodeficiency Research Lab, Centre for Primary Immunodeficiency Ghent, Jeffrey Modell Diagnosis and Research Centre, Ghent University Hospital, Ghent, Belgium; ^3^ Department of Pediatric Nephrology and Rheumatology, Ghent University Hospital, Ghent, Belgium; ^4^ Department of Pediatric Gastroenterology, Hepatology and Nutrition, Ghent University Hospital, Ghent, Belgium; ^5^ Department of Pediatric Endocrinology, Ghent University Hospital, Ghent, Belgium; ^6^ Down Syndrome Clinic, Ghent University Hospital, Ghent, Belgium; ^7^ Department of Pediatric Hematology, Oncology and Stem Cell Transplantation, Ghent University Hospital, Ghent, Belgium; ^8^ Department of Pediatric Neurology and Metabolic Diseases, Ghent University Hospital, Ghent, Belgium

**Keywords:** SARS-CoV-2, COVID-19, serology, tertiary care pediatric patients, chronic diseases

## Abstract

**Background:**

Infection with severe acute respiratory syndrome coronavirus 2 (SARS-CoV-2) is clinically diverse, and children have a low risk of developing severe coronavirus disease 2019 (COVID-19). However, children with chronic diseases have a potentially increased risk.

**Methods:**

We performed a prospective surveillance study with longitudinal serum SARS-CoV-2 anti-nucleocapsid antibody quantification and questionnaires in pediatric tertiary care patients during the first waves of the COVID-19 pandemic (November 2020–September 2021). The results were compared with those of healthy children and adults from the same geographic area.

**Results:**

We obtained 525 samples from 362 patients (M/F ratio of 1.3:1; median age of 11.1 years) comprising children with immune-suppressive or immune-modulating drugs (32.9%), inborn errors of immunity (23.5%), type 1 diabetes mellitus (15.2%), and rheumatic diseases (11.9%). A total of 51 (9.7%) samples were seropositive among 37/351 children (10.5%). Seropositivity increased from 5.8% in November–December 2020 to 21.6% in July–September 2021. Compared with adults, a longitudinal analysis revealed reduced seroprevalence but similar kinetics as in children from the same country. Demographic or social variables and disease characteristics did not correlate with seropositivity. Being obese and household contact with COVID-19-infected individuals significantly increased the odds of infection. The majority of seropositive patients had mild symptoms (21/37). One-third were asymptomatic and/or unaware of having COVID-19 (10/37). Four patients (4/37) needed hospitalization, with good clinical outcomes.

**Conclusions:**

Although harboring a chronic disease, we observed a low SARS-CoV-2 incidence in a cohort of pediatric tertiary care patients, comparable with healthy children during the first year of the pandemic. Infection was mostly associated with mild symptoms.

## 1. Introduction

Infection with severe acute respiratory syndrome coronavirus 2 (SARS-CoV-2) is clinically diverse, ranging from absent or mild symptoms to life-threatening severe coronavirus disease 2019 (COVID-19). The underlying mechanisms and risk factors predisposing to or protecting from severe SARS-CoV-2 infection are only partially explained (1). Children exhibit low susceptibility to severe COVID-19 (2). Despite some immunological hypotheses (3, 4), why young persons, even with underlying chronic diseases, are relatively spared from clinical COVID-19 remains enigmatic.

Reports on COVID-19 in children, especially in those with co-morbidities, have been scarce (5, 6). The majority of studies involving pediatric patients concern retrospective studies or have assessed the prevalence of infection on registry or survey data (5). These reports have suggested a relatively benign disease course of SARS-CoV-2 infection despite the presence of additional risk factors such as primary or secondary immunodeficiency in children (7–12). However, these cohorts of patients were assessed mostly in the very early stages of the pandemic, which explains the low seroprevalence noted (7), or these studies were relatively small in terms of sample size and recruited patients with single underlying diseases (10–12). In addition, most studies had cross-sectional designs and did not evaluate antibody response over time (7–12). Pediatric seroprevalence studies, specifically those with a prospective or longitudinal design, remained limited to date, mainly because of the lack of access to patient data and blood samples (5, 6).

Children undergoing regular follow-ups in tertiary care centers represent a unique research population displaying various chronic diseases with increased susceptibility to infectious diseases. In Belgium, the majority of children with chronic diseases were instructed to comply with strict infection prevention measures during the first waves of the pandemic, including shielding from persons outside the household and not attending school (13). Public health authorities encouraged these stringent measures for children with severe chronic diseases until the summer of 2020.

This study had multiple objectives. First, we aimed to assess COVID-19 incidence in children with underlying chronic diseases. These children may have been less likely to contract SARS-CoV-2 in the first half year of the pandemic because of stringent hygiene measures. With relaxed measures in place and schools reopening after the first lockdown period, to which extent these children were already exposed to SARS-CoV-2 and how incidence would evolve over the coming period remained unclear. Concurrently, we wanted to address questions on the robustness and durability of humoral immune responses in these children. Second, we aimed to study COVID-19 severity since many children with chronic diseases harbor theoretic risk factors for a severe course with infection, such as primary or secondary immune deficiency. Finally, we wanted to assess risk factors associated with SARS-CoV-2 infection to identify which demographic, clinical, and social variables predispose these children to SARS-CoV-2, which could be useful for future preventive techniques. Therefore, we set up a prospective study with longitudinal surveillance of SARS-CoV-2 incidence by monitoring blood serology in children undergoing regular follow-ups in a tertiary care pediatric hospital in Belgium from November 2020 to September 2021.

## 2. Methods

### 2.1. Study participants

Children (0–18 years of age) with chronic diseases that require regular follow-ups in the Princess Elisabeth Children's Hospital (Ghent University Hospital, Belgium) were eligible for inclusion. We defined the regularity of routine follow-ups based on expected follow-up consultations within 6 months. Recruitment occurred in an ambulatory setting with physicians from 10 pediatric departments, namely, Down syndrome clinic, endocrinology, gastroenterology, hemato-oncology, hepatology, immunology, nephrology, neurology, pulmonology, and rheumatology. After parental informed consent and participant assent were obtained, serum blood was collected from children undergoing venipunctures for routine care purposes between 1 November 2020 and 28 February 2021 (baseline sample). Patients sampled in this inclusion period were eligible for follow-up sample collection, with an interval minimum of 3 months and maximum of 6 months. Blood sampling was based on clinical indication as determined by the treating physician, and additional blood volume required for the study was obtained through the same venipuncture procedure. The study was completed on 30 September 2021. Given the explorative and observational design of the study, no sample size was calculated prior to the initiation of the study.

### 2.2. Clinical data collection

During each study visit, the parents completed a questionnaire specifically designed for this study, with topics concerning household characteristics, school attendance, and evidence for SARS-CoV-2 infection in the child or its close contacts, assessed clinically and/or through polymerase chain reaction (PCR) or serology testing. A clinical diagnosis of COVID-19 was defined according to the Belgian national case definition (Sciensano, the Belgian Institute for Health), which included the onset of acute symptoms (cough, dyspnea, thoracic pain, anosmia, or dysgeusia) with at least one other symptom (fever, myalgia, fatigue, rhinitis, throat ache, headache, anorexia, diarrhea, or confusion or fall in the elderly) or unexplained deterioration of chronic respiratory symptoms (asthma, chronic obstructive pulmonary disease, etc.). The physician registered additional data on a standardized form, including demographics, chronic disease, ethnicity, body mass index (BMI), and use of medication affecting immune function. BMI percentiles were calculated for age and sex using the CRAN package *childsds* (v0.8.0) in R, using references for Flemish children (14), or the WHO growth charts for children <2 years old (15). Overweight was defined as BMI between the 85th and 95th percentile, and obesity as BMI greater than or equal to the 95th percentile.

### 2.3. Serological data collection

Anti-SARS-CoV-2 immunoglobulin G targeting the nucleocapsid protein (anti-N) was measured using the enzyme-linked immunosorbent assay (Abbott ARCHITECT 6R86) on the sera of the patients according to the instruction of the manufacturer. Seropositivity was defined as an optical density (OD) ratio ≥0.9 as compared with the calibrator. Due to technical issues (insufficient sample volume or equipment failure), no data were obtained from 11 patients. A physician contacted the parents of patients with positive serology within 1 week after sampling. Clinical symptoms possibly or definitively associated with SARS-CoV-2 infection were registered in detail. Asymptomatic seroconversion was determined when no symptoms were recalled.

We used publicly available data on community seroprevalence rates, as collected by Sciensano, to benchmark the seroconversion rates in our cohort. To have the most relevant comparison, we only used the reported seroprevalence collected from persons living in the same geographic region as our patients and filtered on data collected during the same period as our study. Estimated seroprevalence from healthy children was obtained from longitudinal studies in Belgian schools (16–19), which used a validated serology test in saliva (20). We only used data from pupils from Flemish primary schools to ensure demographic matching with our cohort (these pupils were non-vaccinated during the entire study period). For adults, we used data from the prevaccination era (healthcare workers and blood donors) and from a subcohort of unvaccinated adults, as extracted from two blood-based serology studies (21, 22) and one saliva study (23, 24), respectively.

### 2.4. Statistical analyses

Confidence intervals (CIs) for the proportion of seropositive patients were calculated using the Wilson score interval with a 95% confidence level. To assess risk factors for seroconversion, we used the demographic and clinical variables, social behavior and household characteristics, and exposure to SARS-CoV-2. Odds ratios (OR) were calculated using these variables as predictors. The binary variable, SARS-CoV-2 seropositivity, was used as the outcome. To calculate OR, we used conditional maximum-likelihood estimates. *P*-values were assessed as two-tailed mid *P* Exact. These statistical analyses were performed using OpenEpi (v3.01).

### 2.5. Ethical considerations

The study was approved by the local ethics committee (Ghent University Hospital; BC-08104) and registered on ClinicalTrials.gov (NCT04615000).

## 3. Results

### 3.1. Cohort description

A total of 362 patients were included {with an M/F ratio of 1.3:1; median age 11.1 years [interquartile range (IQR) 7.2–14.6 years]} (Supplementary Table S1). The majority of patients were included in December 2020 (117/362; 32.3%). Progressively fewer patients were recruited in the following months, with a nadir of inclusions by February 2021 (57/362; 15.7%). The majority of patients were Caucasian (295/362; 81.5%), followed by Northern African (22/362; 6.1%) and Middle Eastern/Arab (11/362; 3.0%). The median BMI percentile at inclusion was 51.4 (IQR 21.9–80.6), with 11.4% (40/351) and 11.7% (41/351) of patients having overweight (p85–p95) and obesity (≥p95), respectively.

Patients underwent follow-ups at 10 different pediatric departments, most frequently immunology (25.7%), nephrology (16.9%), and endocrinology (14.4%) (Supplementary Table S2). Various underlying conditions were present, including, most frequently, inborn errors of immunity (IEI) (23.5%), type 1 diabetes mellitus (DM) (15.2%), and rheumatic disease (11.9%). A substantial proportion of patients (119/362; 32.9%) was on immune-suppressive or immune-modulating drugs at the time of sampling (Supplementary Table S3). Tumor necrosis factor inhibitors (37/119; 31.1%), methotrexate (29/119; 24.4%), and tacrolimus (27/119; 22.7%) were most frequently used. Immunoglobulin replacement therapy (IRT) was used in 8.6% (31/362). A total of 23 (6.4%) patients were in a post-transplant setting (liver, *n* = 15; kidney, *n* = 4; hematopoietic stem cell, *n* = 3; and combined liver and kidney, *n* = 1).

Most children (327/339; 97.0%) lived at home, with a median of three household members. At least one of the parents worked outside the home in 86.6% of households (292/337). One-third of the children (117/338; 34.6%) did not attend school or daycare prior to the summer break of 2020. In addition, after the summer break, 36/335 (10.7%) did not go to school or daycare, although no general school closures were applied in Belgium in this period. During follow-ups, a stable proportion of parents declared that their children did not attend school (9.21%).

Patients and parents were questioned about their SARS-CoV-2 exposure during each study visit. The questions included prior infection and/or close contact with infected persons since the start of the pandemic (the first COVID-19 case in Belgium was confirmed on 4 February 2020). The parents noted symptoms suggestive of COVID-19 in 59/362 (16.3%) patients. A PCR had confirmed infection in 26/362 (7.2%). Positive household contacts (assessed by PCR) were present in 51/362 (14.1%), and close contacts outside the household were noted in 48/362 (13.3%).

### 3.2. Serological assessment and characteristics of SARS-CoV-2 infected patients

In total, 525 serum samples were obtained (with an average of 1.45 per patient). The majority (350/525; 66.7%) was collected during the inclusion period (November 2020–February 2021) and one-third (175/525; 33.3%) during follow-ups (March 2021–September 2021). A total of 51 (51/525; 9.7%) samples were positive for SARS-CoV-2 anti-N (95% CI 7.5%–12.6%). On an individual patient level, seropositivity was observed in 37/351 (10.5%; 95% CI 7.7%–14.2%).

Seropositive children had similar demographic and clinical characteristics as the full study population (Supplementary Table S4). In most patients, the parents recalled mild symptoms typically described in COVID-19, such as upper respiratory tract infection and fever (*n* = 21, 56.8%; no data available on two patients). A complete asymptomatic disease was observed in 10 cases (27.0%). Four SARS-CoV-2-positive patients (10.8%) were hospitalized. Two had deterioration of chronic symptoms [electrolyte imbalance and hypertension in a patient with chronic kidney disease (CKD) and dyspnea in a patient with cystic fibrosis (CF)]. One patient with congenital pulmonary aplasia was admitted for overnight observation and monitoring of vital parameters without additional actions. Finally, an infant with pre-existing chronic kidney disease presented protein-losing enteropathy, although it remains unclear if his disease course is attributable to SARS-CoV-2 infection. Besides the patient with CKD and electrolyte imbalance who was hospitalized, 16 other patients on immune-suppressive or immune-modulatory drugs and two patients on subcutaneous immunoglobulins experienced mild respiratory disease at most.

### 3.3. Evolution of seropositivity in the cohort

**Figure 1:**
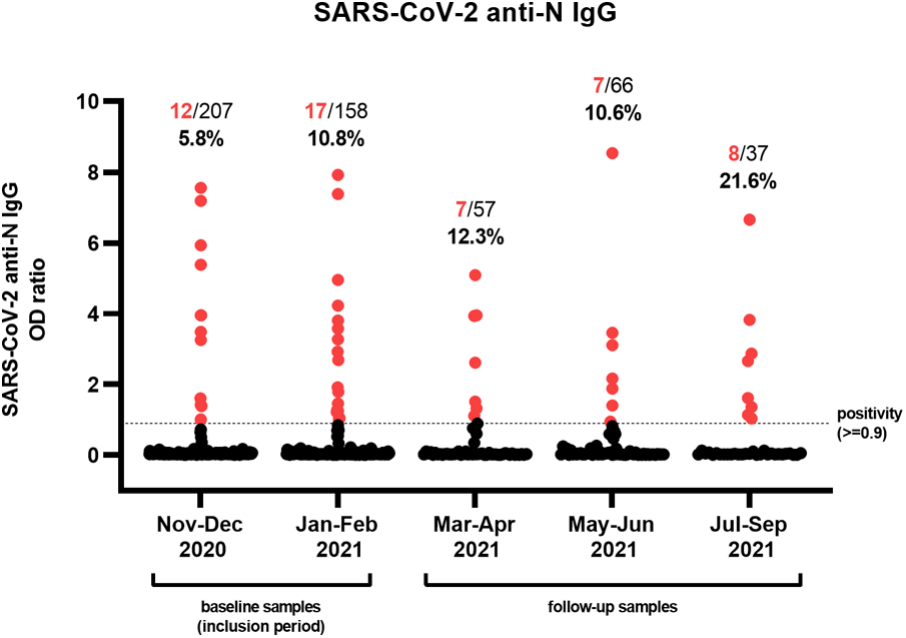
Serological assessment of a cohort of tertiary pediatric patients. Individual samples (*n* = 525) obtained from 362 pediatric patients were analyzed for the presence of antibodies against the nucleocapsid (N) antigen of SARS-CoV-2. The proportion and percentage of serum samples with an OD ratio greater or equal to 0.9 are denoted in red. Patients were included during the first 4 months of the study (November 2020–February 2021), after which they were eligible for follow-up samples until September 2021.

After analyzing seroprevalence longitudinally, the lowest percentage of positive anti-N IgG was observed in the first months of inclusion, with 12/207 (5.8%) positives in November and December 2020 (Figure 1). Although there was less inclusion of samples during the following months, seroprevalence steadily increased in January–February (17/158; 10.8%), March–April (7/57; 12.3%), and May–June 2021 (7/66; 10.6%), with the highest proportion of positive patients found in the last 4 months of sampling (July–September 2021; 8/37; 21.6%).

**Figure 2:**
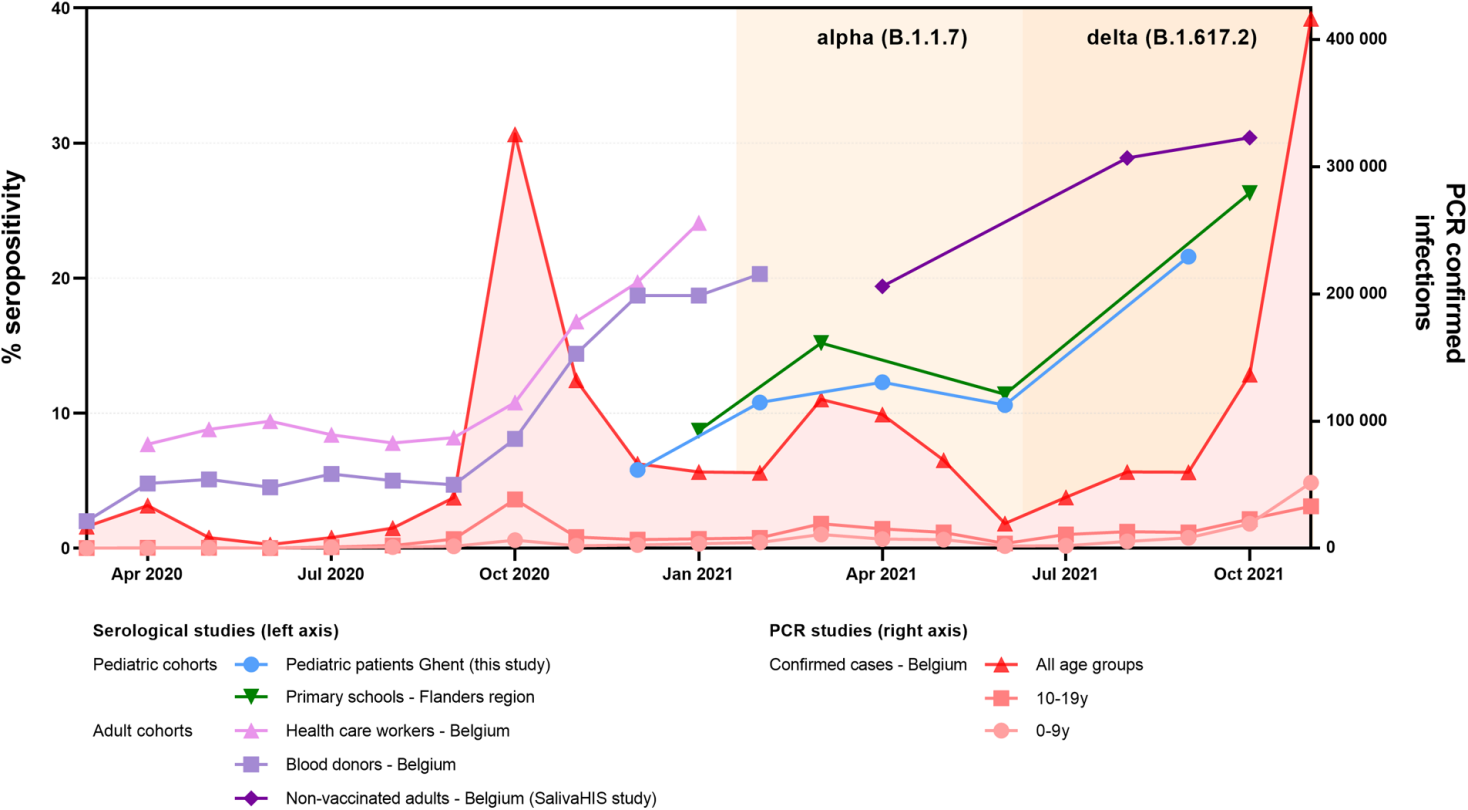
Evolution of percentage of seropositive individuals in this study (blue) as compared with surveillance pediatric and adult populations from the same region/country. National surveillance data based on blood samples from cohorts of healthcare workers (pink) and blood donors (light purple) are plotted until the COVID-19 vaccination campaign was initiated in Belgium (Jan 2021) (21–22). For the adult population, a subcohort of non-vaccinated individuals from another national study monitoring IgG in saliva specimens of healthy adults (SalivaHIS) is shown (dark purple) (23–24). To compare our pediatric data, a surveillance study measuring seroconversion in pupils from schools is plotted (green) (16–19). For this healthy pediatric cohort, we used the readily available and substratified data from the subnational level (Flanders) and concerning children from primary school, to maximally match our cohort of patients. For the epidemiological context, the number of PCR-confirmed infections as counted by the national health institute is mentioned (red, plotted on the right axis) (21). PCR-confirmed infections during the first months of the pandemic should be considered a substantial underestimation (lack of available testing); also, pediatric infections are underrepresented (adapted testing policy from young children in ambulatory setting). In Belgium, for healthy children, schools were re-opened on 15 May 2020. Children with primary or secondary immunodeficiency (considered “high risk” for severe infection) were recommended to stay home longer, with the absence of public recommendations from September 2020 onward.

Our cohort of patients showed reduced seropositivity as opposed to healthy adult populations living in Belgium (Figure 2). In contrast to these adult populations, the seroprevalence of patients largely mirrored the kinetics of healthy children from the same region. Pediatric seroprevalence was prominently reduced as compared with adults prior to alpha (B.1.1.7) dominance, but a sharp increase before and during the first delta (B.1.617.2) wave was noted. Despite these rising numbers, the percentage of positive children remained −5%–10% lower than that in in non-vaccinated adults in Belgium.

**Figure 3:**
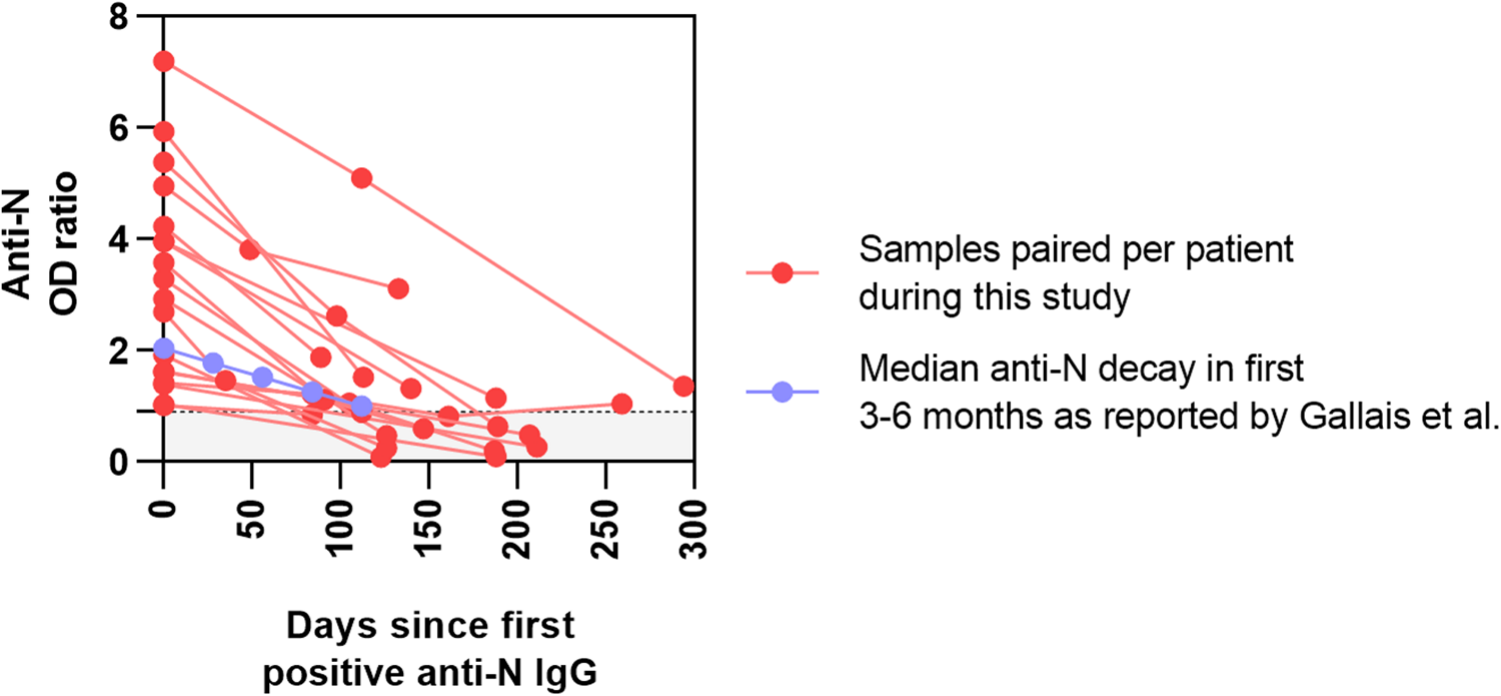
Prospective monitoring of positive samples from our cohort of tertiary pediatric patients. OD ratios of anti-N SARS-CoV-2 serology are plotted in function of days since the first positive test. Paired samples from the patients in this study are shown in red. In blue, the median decay of anti-N in healthy individuals (−0.26/month), as reported by Gallais et al., is presented as a comparator.

Out of the 37 seropositive patients, 18 had at least one additional sample during the study period (Figure 3). From these patients, 26 follow-up samples were obtained (not including the first positive sample), with a median interval of 98 days between samples (range of 35–211 days). The median anti-N decay in this cohort was calculated at 0.42 per month (IQR 0.15–0.61), which is similar to that reported previously (25). Of all follow-up samples obtained within 125 days after the first seropositive sample, 9/12 (75.0%) remained positive, while of those obtained after 125 days, only 5/14 (35.7%) showed persistent seropositivity.

Eight patients who had a positive SARS-CoV-2 PCR did not show seroconversion at the time of sampling. These patients included two patients with low but measurable serum anti-N (OD ratios 0.53–0.67), with mild respiratory disease over 6 months prior to blood sampling. It is plausible that their circulating antibodies had weaned over time. No additional information on the disease course or PCR test could be retrieved for five other patients (OD ratios 0.01–0.07). None had known primary or secondary immune deficiency.

### 3.4. Predictors for seroconversion

**Figure 4:**
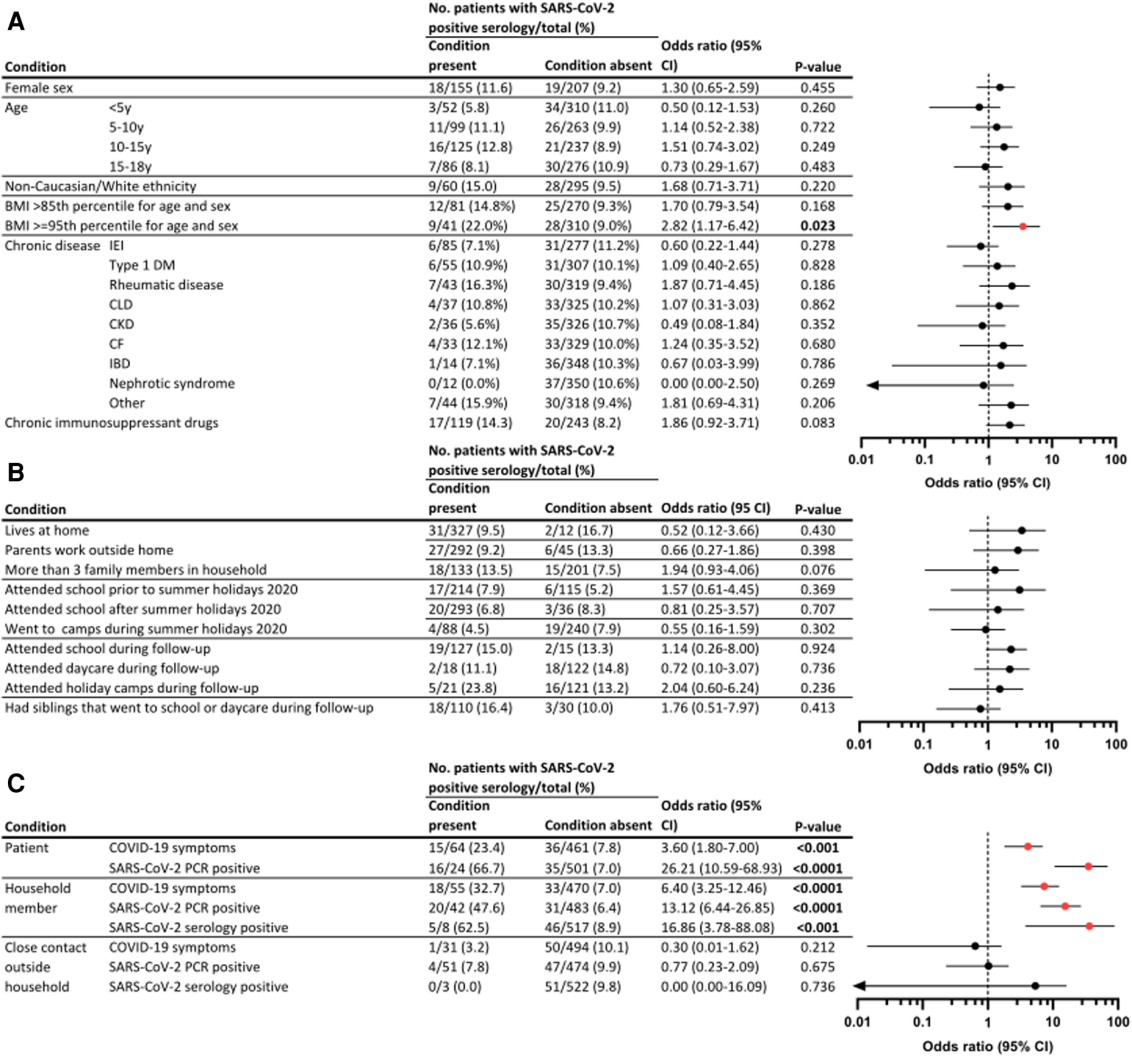
Predictors for SARS-CoV-2 seroconversion in tertiary pediatric patients. Proportion of seropositive patients in this pediatric tertiary cohort, stratified for demographical and clinical variables (**A**), social behavior and household characteristics (**B**), and exposure to SARS-CoV-2 (**C**). ORs and 95% CIs were calculated as conditional maximum-likelihood estimate. *P*-values were assessed as two-tailed mid *P* Exact. Graphs display odds ratios (dot) and 95% CI (line), where significant results are displayed in red. Because of a logarithmic scale, variables including zero are denoted with an arrow. CLD, chronic liver disease.

None of the demographic (sex, age, and ethnicity) parameters were significantly different between those with and without SARS-CoV-2 antibodies (Figure 4A). Among obese patients, we documented a limited increase in odds for seropositivity (OR 2.85, *P* = 0.023), which was not present for patients with overweight (OR 1.70, *P* = 0.168). In contrast, none of the chronic diseases were significantly associated with increased proportions positive for SARS-CoV-2 antibodies. Seroprevalence was largest in patients with rheumatic diseases (7/43; 16.3%) and cystic fibrosis (4/33; 12.1%). Multiple of the largest disease groups (e.g., inflammatory bowel disease (IBD), CKD, and nephrotic syndrome) presented with a maximum of two seropositive patients. Only 2/31 (6.5%) patients on IRT had detectable antibodies throughout the study course. Both were treated using the same commercial subcutaneous Ig product (Hizentra®), showed similarly high OD ratios, and reported no illness. One IRT patient presented an anti-N decay from 3.95 in March 2021 to 1.14 in September 2021. The other IRT patient tested positive (OD ratio 3.83) in August 2021, with a negative sample 6 months before (0.03). From their timelines and the low number of positive patients with IRT, we could, thus, not gather evidence for false reactivity based on immunoglobulin infusions. An increased risk [OR 1.86 (CI 0.92–3.71)] for having positive antibodies was found in patients on chronic immune-suppressive or immune-modulating therapy (17/119) vs. those without such treatment (20/243), although not reaching statistical significance (*P* = 0.083).

Household composition, social behavior, and school attendance did not influence the proportion of patients with antibodies (Figure 4B). Unsurprisingly, when parents declared that COVID-19 symptoms had been present in their child, or if the patient had tested positive using PCR, increased odds for seropositivity were found [OR 3.60 (CI 1.8–7.0); *P* < 0.001 for clinical COVID-19 and OR 26.21 (CI 10.59–68.93); *P* < 0.0001 for PCR positivity] (Figure 4C). In addition, an association with antibody presence was observed if one of the household members experienced a clinical SARS-CoV-2 infection, a positive PCR, or positive serology results, with increased OR of 6.40 (3.25–12.46), 13.12 (6.44–26.85), and 16.86 (3.78–88.08), respectively. Of note, this significant association was not observed when close contacts outside the household were documented with clinical or microbiologically confirmed SARS-CoV-2 infection.

## 4. Discussion

### 4.1. Interpretation of risk factors

By performing a serology-based surveillance study among 362 children with chronic diseases, we describe the incidence and disease severity of COVID-19 in a pediatric tertiary care population during the first year of the SARS-CoV-2 pandemic. Unsurprisingly, patients who were symptomatic, had a positive PCR test, and/or had household contacts with COVID-19 showed an increased risk for seroconversion. A higher risk was not found when children had close contact with infected individuals outside their household, confirming that intimacy of contacts is a major risk factor for SARS-CoV-2 transmission (26–28). We documented a mild increased risk for SARS-CoV-2 antibodies in patients with obesity. While obesity is a well-established risk factor for severe or fatal COVID-19 (29), an association with risk for infection is not typically described and—to our knowledge—not reported in children. Increased seropositivity and a greater magnitude of the antibody response after SARS-CoV-2 infection have been reported in adults with obesity (30–32), while this effect size is not universally replicated (33) and demonstrated to be modulated by the presence or absence of diabetes mellitus (34). In individuals with obesity and metabolic disease, multiple immune-modulatory effects might alter the outcome of viral infection, as extensively reviewed (35). However, given the limited increase in relative risk and cohort size, our data should be interpreted with caution.

Besides obesity, we could not identify additional risk factors, despite the registration of multiple demographic, clinical, and social variables. In particular, primary or secondary immunosuppression was not associated with altered odds for positive serology when compared with immunocompetent patients. Finally, the durability of serological response in our patients seemed comparable to that of healthy individuals (25), despite low numbers of patients with follow-up samples. SARS-CoV-2-specific antibodies have been detected in healthy donor plasma pools from September 2020 onward (36), and cross-reactive antibodies have been identified in commercial Ig products (37). Among our 31 patients on IRT, we only found two seropositive patients. Although both had asymptomatic seroconversion, the timeline and results of other samples during the study did not suggest false reactivity attributable to IRT. Clinicians should remain cautious when interpreting serological testing in patients on IRT.

### 4.2. Interpretation of seroprevalence over time

This study was initiated near the end of the first year of the pandemic. At that time, no substantial sanitary measures had remained in place for children in Belgium (e.g., strict lockdown or school closures were no longer in place). Notwithstanding, by comparing pre-vaccinated and unvaccinated adult populations from the same region, we provide evidence for reduced incidence of pediatric infections during the first year of the pandemic. We hypothesize that children with chronic diseases and their parents might be more restrictive in their social contacts and more rigid in their preventive hygienic measures. The reduced percentage of seropositive children remained present until the third wave of (delta dominant) infections in Belgium, although public sanitary measures were largely relaxed, and no specific instructions remained in place for our patients. Confirmation of delayed incidence of infection in other pediatric studies may be important for future decision-making, not least concerning preventive and hygienic strategies for children with chronic diseases exposed to pandemic viruses.

### 4.3. Strengths of the study

By analyzing a large number of blood samples in children in regular follow-ups at a tertiary care center, we had the unique opportunity to corroborate the presence of circulating antibodies after confirmed or presumed infection and to study antibody evolution over time. In addition, by using serology assays, we most likely captured all previously infected children, including incidental seroconversion in patients with asymptomatic and mild disease. In a context with little data on pediatric COVID-19 because of the lack of PCR testing and impaired access to patient samples (e.g., because of lockdown measures), being able to collect over 500 blood samples from this population is one of the strengths of this study.

### 4.4. Limitations of the study

Our study has multiple limitations. Most importantly, surveillance studies based on serological assays have a high risk of false negatives (antibody decay or severe antibody deficiency). However, measuring antibodies over time allows for comparability with similar studies in the community, as described above. A selection bias could exist since we only included children with regular blood sampling for clinical purposes. Equally, varying preparedness of patients or parents to participate might have been present. With the broad inclusion and few exclusion criteria, we had no mechanisms in place to prevent possible differences in screening for eligibility, recruitment strategy, and motivation to include patients among study personnel. We also had the most samples in the first months, with declining numbers thereafter. This decline in samples included missed samples from patients lost to follow-up, patients that unexpectedly had no blood samples performed, or requesting clinicians that were (no longer) aware that their patient was included in the study. Given the observational design of the study and due to relatively low seropositivity, we were underpowered to fully probe the serological response upon infection or assess antibody decline over time in our patients. Symptoms were requested in retrospect, which might have introduced bias toward reporting severe disease and/or not recalling mild or atypical disease. Finally, in a substantial proportion of patients reporting positive PCR, we were unable to access the test result or document details on the disease course. As an expertise center, we care for children living in a broad region in Belgium. However, we acknowledge that the data presented here are best regarded as a single-center experience, and our findings may not be extrapolated to other pediatric cohorts.

## 5. Conclusions

Our data confirm that children with chronic diseases undergoing follow-ups at a pediatric tertiary care center represent a distinct population and that their exposure to pandemic viruses can be reduced as opposed to adults. Observations made in the community in terms of epidemiology, disease course, and prognosis might not be translatable to these populations. Although we could confirm that vulnerable children, such as those immunocompromised, most often display mild COVID-19, seroprevalence was low to absent in some particular populations, even after more than 1 year of viral circulation in the community. Further unraveling of factors impacting disease severity will be crucial in the future. Insights in COVID-19 pathogenesis and clinical impact, both in those affected and spared from severe disease, will not only allow for treatment optimization but also rationalize preventive actions such as shielding and vaccination strategies. By documenting good clinical outcomes in this cohort, we advocate for nuanced protective and preventive measures and suggest additional research efforts to propagate knowledge and rationalize management concerning COVID-19 in children with chronic diseases.

## Data Availability

The raw data supporting the conclusions of this article will be made available by the authors, without undue reservation.
