# Supplementary material for: Comparison of SARS-CoV-2 seroconversion in children with chronic diseases with healthy children and adults during the first waves of the COVID-19 pandemic
Source: Front Pediatr. 2023 Aug 7;11:1210181. doi: 10.3389/fped.2023.1210181 (PMC10440688; doi:10.3389/fped.2023.1210181)
Supplement: Supplementary file 1 [file Table1.docx]

Supplementary Material

SARS-CoV-2 seroconversion in children with chronic diseases compared with healthy children and adults during the first waves of the COVID-19 pandemic

Hoste Levi^1,2^, Prytula Agniezska^3^, Dehoorne Joke^3^, De Bruyne Ruth^4^, Van Biervliet Stephanie^4^, De Waele Kathleen^5^, Maes Evelyn^6^, Bordon Victoria^7^, Vanlander Arnaud^8^, Claes Karlien^2^, Vande Walle Johan^3^, Schelstraete Petra^1^, Van daele Sabine^1^, Haerynck Filomeen^1,2*^

*** Correspondence:**Filomeen Haerynck, Department of Pediatric Pulmonology, Infectious Diseases and Immunology, Ghent University Hospital, Corneel Heymanslaan 10, Ghent, Belgium, filomeen.haerynck@ugent.be

# Supplementary Tables

**Supplementary Table 1:** Demographic characterization of the cohort

|  | **Study cohort (n=362)** |
| --- | --- |
| Demographics |  |
| Female sex, n (%) | 155 (42.8) |
| Age, median (IQR) | 11.1y (7.2-4.6) |
| Age group |  |
| *<5yo* | *52 (14.4)* |
| *5-10yo* | *99 (27.3)* |
| *10-15yo* | *125 (34.5)* |
| *15-18yo* | *86 (23.8)* |
| Ethnicity, n (%) |  |
| *White/Caucasian* | *295 (81.5%)* |
| *Northern African* | *22 (6.1%)* |
| *Middle Eastern/Arab* | *11 (3.0%)* |
| *Black/African* | *9 (2.5%)* |
| *Other* | *10 (2.8%)* |
| *Unknown* | *15 (4.1%)* |
| Antropometry |  |
| BMI, median (IQR) | 17.6 kg/m² (15.7-20.3) |
| BMI percentile, median (IQR) | 51.4 (21.9-80.6) |
| Overweight (BMI >85^th^ and <95^th^ percentile for age and sex), n (%) | 40/351 (11.4) |
| Obese (BMI >= 95^th^ percentile for age and sex), n (%) | 41/351 (11.7) |
| Serum samples obtained for serological study |  |
| Number of samples, n | 525 |
| Number of patients with more than 1 sample, n (%) | 188 (51.9) |

Percentages are calculated on the total cohort (n=362), unless otherwise stated in case of missing data. BMI: body mass index, IQR: interquartile range

**Supplementary Table 2:** Clinical characterization of the cohort

|  | **Study cohort (n=362)**  **n (%)** |
| --- | --- |
| **Pediatric department of active follow-up** |  |
| Immunology | 93 (25.7) |
| Nephrology | 61 (16.9) |
| Endocrinology | 52 (14.4) |
| Rheumatology | 41 (11.3) |
| Pulmonology | 35 (9.7) |
| Hepatology | 28 (7.7) |
| Gastroenterology | 22 (6.1) |
| Neurology | 11 (3.0) |
| Hemato-oncology | 6 (1.7) |
| Down syndrome | 4 (1.1) |
| Uncategorized | 9 (2.5) |
| **Chronic diseases/reasons for follow-up** |  |
| Inborn error of immunity (IEI) | 85 (23.5) |
| *Predominantly antibody deficiency* | *51 (14.1)* |
| *Other IEI* | *34 (9.4)* |
| Type 1 diabetes mellitus | 55 (15.2) |
| Rheumatic disease | 43 (11.9) |
| *(s)JIA* | *31 (8.6)* |
| *Other rheumatic disease* | *12 (3.3)* |
| Chronic liver disease (CLD) | 37 (10.2) |
| *Post-liver transplantation* | *16 (4.4)* |
| *Autoimmune hepatitis* | *8 (2.2)* |
| *CLD not further specified* | *13 (3.6)* |
| Chronic kidney disease (CKD) | 36 (9.9) |
| *Post-kidney transplantation* | *4 (1.1)* |
| *Atypical hemolytic uremic syndrome* | *4 (1.1)* |
| *ADPKD/ARPKD* | *3 (0.8)* |
| *CKD not further specified* | *25 (6.9)* |
| Cystic fibrosis | 33 (9.1) |
| Inflammatory bowel disease | 14 (3.9) |
| Nephrotic syndrome | 12 (3.3) |
| Other chronic diseases (affecting less than 2% of the cohort) | 44 (12.2) |
| *Epilepsy* | *7 (1.9)* |
| *Acute lymphatic leukemia* | *6 (1.7)* |
| *Down syndrome* | *6 (1.7)* |
| *CAKUT* | *5 (1.4)* |
| *Other kidney disorder* | *5 (1.4)* |
| *Undifferentiated immune disorder* | *4 (1.1)* |
| *Benign hematologic disorder* | *3 (0.8)* |
| *Other respiratory disorder* | *3 (0.8)* |
| *Other endocrine disorder* | *2 (0.6)* |
| *Other gastrointestinal disorder* | *2 (0.6)* |
| *Other nervous system disorder* | *1 (0.3)* |
| Uncategorized | 3 (0.8) |

Percentages are calculated on the total cohort (n=362)

**Supplementary Table 3:** Therapeutics used in the cohort

|  | **Study cohort (n=362)**  n (%) |
| --- | --- |
| Immune suppressing or modulating therapy | 119 (32.9) |
| Tumor necrosis factor inhibitors | 37 (10.2) |
| Tacrolimus | 27 (7.5) |
| Methotrexate | 29 (8.0) |
| Mycophenolic acid | 17 (4.7) |
| Azathioprine | 15 (4.1) |
| Systemic steroids | 18 (5.0) |
| 6-Mercaptopurine | 6 (1.7) |
| Hydroxychloroquine | 6 (1.7) |
| Sirolimus | 4 (1.1) |
| Abatacept | 3 (0.8) |
| Ciclosporin | 2 (0.6) |
| Interleukin-6 inhibitors | 2 (0.6) |
| Hydroxycarbamide | 2 (0.6) |
| Colchicine | 2 (0.6) |
| Vedolizumab | 1 (0.3) |
| Tofacitinib | 1 (0.3) |
| Eculizumab | 1 (0.3) |
| Rituximab | 1 (0.3) |
| Immunoglobulin replacement therapy | 31 (8.6) |
| Post-transplant setting | 23 (6.4) |
| Liver transplantation | 15 (4.1) |
| Kidney transplantation | 4 (1.1) |
| Hematopoietic stem cell transplantation | 3 (0.8) |
| Combined liver and kidney transplantation | 1 (0.3) |

Percentages are calculated on the total cohort (n=362)

**Supplementary Table 4:** Characteristics of SARS-CoV-2 seropositive patients

| **Case** | **SARS-CoV-2 IgG titer** | **Sex** | **Age (y)** | **BMI (kg/m²)**  **(percentile for age and sex)** | **Chronic disease (or reason for follow-up)** | **Immune suppressing or modulating drugs or IRT** | **Symptom description** | **Hospital admission** | **PCR+** | **Presumed source of infection** |
| --- | --- | --- | --- | --- | --- | --- | --- | --- | --- | --- |
| **1** | 1.39 | M | 5 | 14.54 (0.20) | FMF | Colchicine | Intermittent fever attributed to underlying disease |  | No | Unknown, mother seropositive too |
| **2** | 1.61 | F | 8 | 13.20 (0.03) | oJIA | MTX | URTI - 1 day |  | No | Family |
| **3** | 1.01 | F | 13 | 30.04 (0.99) | oJIA, Turner syndrome | Infliximab | None |  | No | Unknown |
| **4** | 3.95 | M | 9 | 23.47 (0.99) | sJIA | Tocilizumab | Intermittent fever attributed to underlying disease |  | No | Unknown |
| **5** | 7.19 | M | 6 | 18.49 (0.96) | Selective polysaccharide antibody deficiency |  | Multiple URTI with fever over last months |  | No | Unknown |
| **6** | 3.26 | M | 14 | 17.79 (0.33) | CKD | Tacrolimus, MPA | Deterioration of chronic symptoms | Yes, electrolyte disturbance and hypertension | Yes | Unknown |
| **7** | 3.95 | M | 15 | 28.81 (0.98) | Ciliopathy-associated liver disease |  | Fever, cough, fatigue - 2 days; anosmia 7 days |  | No | Family |
| **8** | 5.93 | F | 9 | 14.00 (0.08) | CF |  | Fever, cough, thoracal pain - 1 week | Yes, borderline hypotension and dyspnea | Yes | Family |
| **9** | 1.42 | M | 3 | 19.86 (0.99) | CF |  | Fever, cough - 1 day |  | No | Unknown |
| **10** | 5.38 | M | 10 | 16.39 (0.46) | DM type 1 |  | None |  | No | Unknown |
| **11** | 3.49 | M | 16 | 27.54 (0.96) | DM type 1 |  | URTI, fatigue - 1 day |  | Yes | Leisure/sports outside school |
| **12** | 7.56 | F | 12 | 21.12 (0.82) | DM type 1 |  | URTI, fatigue, higher glucose levels - 2 days |  | Yes | Family |
| **13** | 4.95 | F | 15 | 33.69 (1.00) | Asthma |  | Dyspnea, less control of asthma, fatigue - 7 days |  | Yes | School |
| **14** | 3.28 | M | 10 | 14.64 (0.11) | Sickle cell anemia | Hydroxycarbamide | URTI, 3-4 days |  | No | Family |
| **15** | 7.38 | F | 14 | 18.95 (0.42) | pJIA | MTX | None |  | No | Family |
| **16** | 1.25 | M | 9 | 17.39 (0.73) | Sickle cell anemia | Hydroxycarbamide | None |  | No | Family |
| **17** | 2.93 | F | 11 | 18.11 (0.59) | CAH | HC substitution dose | URTI, fatigue, headache - 3-4 days |  | Yes | Unknown |
| **18** | 2.69 | M | 17 | 17.72 (0.08) | CRMO | Infliximab | Fever, fatigue - 3-4 days; thoracal pain, diarrhea - 1 day |  | Yes | Unknown |
| **19** | 3.58 | M | 11 | 16.68 (0.44) | CAH, asthma | HC substitution dose | Headache, fatigue - 1 day |  | No | Unknown |
| **20** | 7.92 | F | 8 | 13.42 (0.05) | Congenital pulmonary dysplasia |  | Fever, URTI, headache, diarrhea - 1 day | Yes, 1 day observation | Yes | Family |
| **21** | 1.03 | F | 12 | 16.72 (0.29) | CF |  | Headache, fatigue - 3-4 days |  | No | Unknown |
| **22** | 4.23 | F | 16 | 23.87 (0.82) | DM type 1 |  | URTI - 1 day |  | No | Family |
| **23** | 1.79 | F | 18 | 20.80 (0.42) | oJIA | Etanercept | URTI, anosmia, fatigue - 10 days |  | No | Family |
| **24** | 1.29 | F | 14 | 17.70 (0.23) | Caroli disease, post-LKTX | Tacrolimus, MPA | None |  | No | Family |
| **25** | 1.92 | M | 0.6 | 16.20 (0.20) | ARPKD |  | Severe protein loosing enteropathy | Yes, ICU  SARS-CoV-2 infection was presumed to be unrelated to the enteropathy (incidental positive) | Yes | Family |
| **26** | 3.94 | F | 14 | 25.45 (0.94) | IBD | Vedolizumab | Fever - 1-2 days |  | Yes | Family |
| **27** | 3.95 | M | 12 | 18.84 (0.68) | Hypogamma- globulinemia | SCIG | None |  | No | Unknown |
| **28** | 1.41 | F | 11 | 16.38 (0.32) | CF |  | None |  | No | Unknown |
| **29** | 8.54 | M | 5 | 18.41 (0.96) | CKD |  | Rhinitis, low grade fever - 3-4 days |  | Yes | Family |
| **30** | 0.95 | M | 15 | 18.66 (0.36) | Post-LTX, refractory cytopenia of childhood | Ciclosporin | Fever, cough – 1 day |  | Yes | Unknown |
| **31** | 3.46 | F | 10 | 14.44 (0.10) | pJIA | Etanercept | NA |  | Yes | Unknown |
| **32** | 2.17 | F | 16 | 29.75 (0.98) | DM type 1 |  | None |  | No | Unknown |
| **33** | 6.66 | M | 15 | 18.02 (0.26) | DM type 1 |  | None |  | Yes | Family |
| **34** | 1.62 | F | 15 | 16.63 (0.05) | STAT1 gain-of-function |  | Loss of taste and smell |  | Yes | Family |
| **35** | 2.66 | M | 1 | 18.9 (0.92) | Down syndrome |  | NA |  | No | Unknown |
| **36** | 3.83 | M | 8 | 18.15 (0.87) | Hypogamma- globulinemia | SCIG | None |  | No | School |
| **37** | 2.87 | F | 9 | 14.81 (0.20) | JIA | Adalimumab | Headache, abdominal pain |  | Yes | Leisure/sports outside school |

Supplementary Table 4: Clinical characteristics and COVID-19 related symptoms of the 37 seropositive patients among a cohort of 362 pediatric patients. Immunoglobulin G (IgG) titer represents the optical density ratio as compared to the calibrator of an anti-nucleocapsid enzyme-linked immunosorbent antibody assay. Abbreviations used: ARPKD autosomal recessive polycystic kidney disease, BMI body mass index, CAH congenital adrenal hyperplasia, CF cystic fibrosis, CKD chronic kidney disease, CRMO chronic recurrent multifocal osteomyelitis, DM diabetes mellitus, FMF familial Mediterranean fever, HC hydrocortisone, ICU intensive care unit, JIA juvenile idiopathic arthritis, L(K)TX liver (and kidney) transplantation, MPA mycophenolic acid, MTX methotrexate, oJIA oligo-articular JIA, pJIA pauci-articular JIA, PsA psoriasis arthritis, PCR+ polymerase chain reaction positive for SARS-CoV-2, pJIA polyarticular JIA, sJIA systemic JIA, URTI upper respiratory tract infection.
